# Supplementary material for: Antioxidant Activity and Photosynthesis Efficiency in Melissa officinalis Subjected to Heavy Metals Stress
Source: Molecules. 2023 Mar 14;28(6):2642. doi: 10.3390/molecules28062642 (PMC10053282; doi:10.3390/molecules28062642)
Supplement: Supplementary file 1 [file molecules-28-02642-s001.zip › molecules-2241767-supplementary.pdf]

Antioxidant activity and photosynthesis efficiency in *Melissa Officinalis* subjected to heavy metals stress

Dorota Adamczyk-Szabela[a*], Ewa Chrześcijańska [a], Piotr Zielenkiewicz [b], Wojciech M.Wolf [a]

[1] *Technical University of Lodz, Institute of General and Ecological Chemistry, Faculty of Chemistry, Zeromskiego 116, 90-924 Lodz, Poland*

[2] *Institute of Biochemistry and Biophysics Polish Academy of Sciences Department of Bioinformatics, Pawińskiego 5a; 02-106 Warsaw, Poland*

Corresponding author: Adamczyk-Szabela Dorota [a*], e-mail: dorota.adamczyk@p.lodz.pl

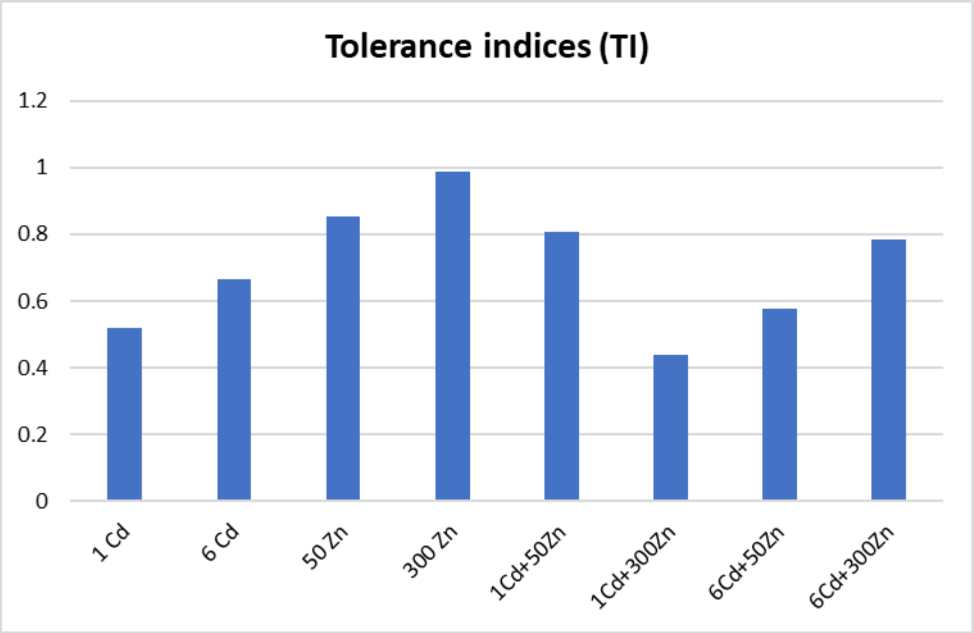

Figure S1. Tolerance indices (TI) calculated for all Cd and Zn treatments

Table S1. Potentials, currents, partial and total activities as determined by CV (a) and DPV (b) methods

(a)

No	Samples	Peak number	Potential (V)	Current (μA)	Partial activity (μA/V)	Total activity (μA/V)	Order in terms of activity
1	Control	1	1.26	25.0	20.0	**71.0**	**9**
		2	1.91	97.0	51.0		
2	1Cd	1	1.19	22.0	19.0	**80.0**	**8**
		2	1.94	118	61.0		
3	6Cd	1	1.91	198	104	**104**	**6**
4	50Zn	1	1.15	21.0	18.0	**95.0**	**7**
		2	1.67	55.0	33.0		
		3	1.96	86.0	44.0		
5	300Zn	1	0.98	17.0	17.0	**119**	**5**
		2	1.59	72.0	45.0		
		3	1.96	111	57.0		
6	1Cd+50Zn	1	0.90	17.0	19.0	**123**	**4**
		2	1.41	64.0	46.0		
		3	1.91	110	58.0		
7	1Cd+ 300Zn	1	0.95	14.0	15.0	**124**	**3**
		2	1.56	68.0	43.0		
		3	1.88	123	66.0		
8	6Cd+50Zn	1	1.22	24.0	19.0	**154**	**2**
		2	1.64	81.0	50.0		
		3	1.91	161	85.0		
9	6Cd+ 300Zn	1	0.28	14.0	49.0	**251**	**1**
		2	0.68	23.0	34.0		
		3	1.66	113	68.0		
		4	1.95	196	100		

(b)

No	Samples	Peak number	Potential (V)	Current (µA)	Activity (µA/V)	Total activity (µA/V)	Order in terms of activity
1	Control	1	0.79	2.42	3.06	**6.39**	**9**
		2	1.96	6.51	3.33		
2	1Cd	1	0.87	2.61	2.99	**6.63**	**8**
		2	1.99	7.22	3.64		
3	6Cd	1	0.24	2.20	9.36	**43.3**	**2**
		2	0.39	2.37	6.14		
		3	0.60	3.67	6.09		
		4	1.84	39.9	21.7		
4	50Zn	1	0.84	2.72	3.24	**11.0**	**7**
		2	1.66	5.15	3.11		
		3	1.88	8.74	4.65		
5	300Zn	1	0.87	3.24	3.75	**11.4**	**6**
		2	1.57	5.07	3.24		
		3	1.85	8.22	4.45		
6	1Cd+50Zn	1	0.90	3.71	4.15	**11.5**	**5**
		2	1.62	4.99	3.08		
		3	1.82	7.68	4.23		
7	1Cd+300Zn	1	0.92	2.54	2.78	**18.8**	**4**
		2	1.84	29.4	16.0		
8	6Cd+50Zn	1	1.54	4.10	2.66	**21.5**	**3**
			1.90	35.8	18.9		
9	6Cd+300Zn	1	0.23	10.7	45.9	**78.0**	**1**
		2	0.46	3.67	7.99		
		3	0.60	4.88	8.20		
		4	1.64	9.47	5.78		
		5	1.84	18.7	10.2		

Table S2. Metals content in the certified reference material (p = 0.95, n = 6).

**Metal**	**Certified value µg/g**	**Found µg/g**	**Recovery %**
**Cadmium**	0.199 ± 0.015	0.195 ± 0.010	98
**Zinc**	33.5 ± 2.1	34.2 ± 1.6	102
**Copper**	7.77 ± 0.53	7.65 ± 0.49	98
**Manganese**	191 ± 12	184 ± 10	96
